# Relationship between dental anxiety levels and oral health among dental patients in Turkey: a cross-sectional study

**DOI:** 10.1186/s12903-023-03041-8

**Published:** 2023-05-25

**Authors:** Zafer Saba, Gunseli Katirci

**Affiliations:** 1 Restorative Dentistry Specialist, Private Dentist, Bursa, Turkey; 2 Faculty of Dentistry, Department of Restorative Dentistry, Suleyman Demirel University, Isparta, Turkey

**Keywords:** Dental anxiety, MDAS, Gingival index, Dental caries, DMFT, Oral–dental health

## Abstract

**Background:**

This study aimed to determine the relationship between dental anxiety and oral health in adult patients who applied to the Department of Restorative Dentistry at the Faculty of Dentistry at Suleyman Demirel University.

**Methods:**

The study included 500 subjects. The dental anxiety levels of the patients were determined using a modified dental anxiety scale (MDAS). Information on sociodemographic details, oral hygiene and nutritional habits were recorded. Intraoral examinations of the subjects were performed. Caries prevalence of individuals was determined using the decayed, missing or filled teeth (DMFT) and decayed, missing, or filled surfaces (DMFS) indices. Gingival health was evaluated using the gingival index (GI). Statistical analyses were performed using the Mann–Whitney U, Kruskal–Wallis and Chi-square tests and Spearman correlation analysis.

**Results:**

The ages of the 276 female and 224 male participants ranged from 18–84 years. The median MDAS value was 9.00. The median DMFT and DMFS values were 10.00 and 23.00, respectively. The median MDAS values ​​of women were higher than those of men. Individuals who postponed their appointment had a higher MDAS median value than those who did not (Mann–Whitney U test, *p* < 0.05). No statistically significant correlation was found between dental anxiety level (MDAS) and GI, DMFT and DMFS index scores (Spearman correlation analysis, *p* > 0.05).

**Conclusion:**

The MDAS values of individuals who did not remember the reason for their dental visit were higher than those who visited the dentist for routine control. Based on the findings of this study, further research on the relationship between dental anxiety and oral health is necessary to determine the factors that pose a risk for dental anxiety and to ensure the regular benefits of dental services.

## Introduction

Dental anxiety is widespread and poses an important problem during the management of patients in different age groups [1, 2]. In developed countries, patients are affected by dental anxiety [3]. Dental hygiene habits are affected by dental fear [4]. Studies have shown that patients with dental fear commonly ignore dental care, and dental anxiety can be an important cause of missed or cancelled dental appointments [1, 3].

Dental anxiety is a multifactorial condition [3]. Previous dental experiences play a major role in developing dental anxiety, which is exhibited by painful and traumatic experiences regarding dental treatment, unpleasant dentist contact and general fearfulness [5, 6]. Enhancing dental anxiety includes fear of pain, introversion, weakness, loss of control, fear of unexpected events and shame [7]. Children with parents with dental anxiety can be affected and are highly prone to dental fear [8]. Studies have shown that the prevalence of dental anxiety ranges from 11% to 91.2%. These differences can be attributed to the subjectivity of the diagnostic criteria, assessment methods and sampling techniques of the studies. A study has reported a high prevalence of dental anxiety in a group of adult patients in India at 46% [9]. Other studies have reported that the prevalence rates of dental anxiety are at 61.3%, 72% and 91.2% [10–12]. In Turkey, the reported prevalence rates of dental anxiety are at 5.1%, 21.3-23.5%, and 42.1% [13–15].

A vicious cycle exists between dental anxiety, which leads to delayed dental care, and oral health, which causes dental deterioration. Individuals entering this vicious circle may have increased dental anxiety and may require more dental treatment for an existing oral problem than persons who routinely visit the dentist for regular control [16, 17]. In addition, a great functional disturbance may occur in patients with dental anxiety, and their quality of life can decrease [18, 19]. This condition can damage their psychological affluence, social function and vitality. Several studies have reported that dental anxiety causes poor oral health, with more decayed, missing and fewer filled teeth and worse periodontal health [18, 20, 21]. The oral health of patients with dental anxiety still has inconclusive findings [3, 21]. At present, the public’s dental anxiety should be evaluated, as this causes management problems during dental treatment and to determine its relationship with oral health status to improve the condition [12].

This study aimed to determine the relationship between dental anxiety and sociodemographic factors and oral health involving dental and periodontal status in patients attending the Suleyman Demirel University in Isparta, Turkey.

## Materials and methods

### Ethics

The present cross-sectional study was performed in the clinics of the Restorative Dentistry Department at the Faculty of Dentistry at Suleyman Demirel University. The ethics committee of Suleyman Demirel University, Faculty of Medicine, verified the study protocol (SDU.05.05.2021-11/193) and written informed consent was acquired from each patient before the study. The present study was conducted from May 2021 to August 2021.

### Sample

G-Power software package programme (G*Power Ver. 3.0.10, Germany) was used for the power analysis. At least 459 patients were essential for the study, with 90% power in the *d* = 0.15 impact width. To avoid possible data loss, we added 41 patients and included a total of 500 patients in the study. Adult patients aged between 18 and 84 years and without any physical or mental illness were included in the study.

All patients were reported with a standardised structured questionnaire. The questionnaire provided information regarding sociodemographic characteristics, oral care, dental visits, parafunctions, smoking habits, diet, postponing dental appointment habits, dental fillings and painful experiences. The questionnaires were immediately completed under supervision.

### Intraoral examination

The intraoral dental examination of all patients was performed by the same dentist using a conventional dental probe, mirror, and overhead dental lamp**.** Digital panoramic radiographs (Planmeca, Helsinki, Finland) were used to detect proximal caries lesions. Bite-wing radiographs were taken in addition to panoramic radiographs in cases where interproximal contact areas could not be recognized alone due to superpositions.

The total number of teeth, decayed, missing, or filled teeth (DMFT), decayed, missing, or filled surfaces (DMFS) and gingival index (GI) scores were determined. After air drying the teeth, the DMFT and DMFS scores were calculated. Caries were identified according to the criteria of the World Health Organization (WHO 1997) [22]. Teeth with a carious lesion in a pit or fissure, or on the tooth surface, and teeth with a softened floor, softened wall, or undermined enamel are recorded as carious. A filled tooth containing decayed areas was also recorded as carious. The restored teeth due to caries were recorded as filling. The extracted teeth due to caries were recorded as missed. Teeth restored for causes other than caries were not counted as filling. Third molars were not included in the current study. Periodontal status was identified according to GI. The six index teeth (i.e. teeth 44, 32, 36, 24, 12 and 16) were assessed with GI of Silness and Löe [23].

### Dental anxiety score

A modified dental anxiety scale (MDAS) was used to evaluate dental anxiety in the current study [24]. The scale is comprised of five questions to measure the anxiety level in a certain dental condition before the clinical examination. The scale consists of the following questions: (Q1) If you had to visit the dentist tomorrow, how would you feel about it? (Q2) While waiting for your turn in the dental office, how would you feel? (Q3) How would you feel if you knew that a tooth drill would be used? (Q4) How would you feel if you knew that your teeth would be scaled and polished? (Q5) How would you feel if you knew that a local anaesthetic injection would be administered in your gingiva or over an upper back tooth?

The answers were calculated based on the MDAS, and ratings of 1–5 demonstrated *not anxious*, *slightly anxious*, *fairly anxious*, *very anxious,* and *extremely anxious*, correspondingly. Therefore, the maximum score that can be calculated from each question was 5. In addition, the minimum and maximum scores of the entire scale were rated 5 and 25, respectively.

MDAS scores of 5–9, 10–18, and ≥19 were defined as less anxiety, moderate anxiety, and high dental anxiety, respectively [25].

### Statistical analysis

The statistical program SPSS (version 25.0) was used to evaluate the data. The normality distribution of the data was checked using the Shapiro–Wilk and Kolmogorov–Smirnov tests. The relationships between the MDAS and categorical variables were determined using the Chi-square test. The relationships between the MDAS and GI scores were evaluated using the Chi-square test. Group differences were analysed using the Mann–Whitney U or Kruskal–Wallis tests and Bonferroni post hoc correction, as necessary. The correlation between the scores of the MDAS and DMFT or DMFS indexes were assessed with Spearman’s correlation analysis. The level of significance was 0.05 for each statistical analysis.

## Results

The present study included 500 participants, with a median MDAS value of 9.00 (Table 1). The Cronbach’s alpha of the MDAS was 0.898 for the current study, which indicated good internal consistency and reliability (*N* = 500). Furthermore, 51 (10.2%) of the 500 patients may be categorized with a high MDAS score of 19 or greater (Table 2).

**Table 1:** Median score of the decayed, missing or filled teeth (DMFT), decayed, missing, or filled surfaces (DMFS) and modified dental anxiety scale (MDAS) scores of the study participants (*N* = 500)

**Variable**	**Median**	**Minimum–Maximum Value**
DMFT	10	1.00–30.00
DMFS	23	1.00–126.00
MDAS	9	5.00–25.00

**Table 2:** Distribution of individuals in the study according to their dental anxiety level (*N* = 500)

**Modified Dental Anxiety Scale Score**	***N***	**%**
5–9 (*less anxiety*)	262	52.4
10–18 (*moderate anxiety*)	187	37.4
≥19 (*high anxiety*)	51	10.2

The median MDAS value of patients in the age group of 20–34 years was higher than that of the individuals in the age group of 50–64 years (Kruskal–Wallis test, *p* < 0.05). A significant relationship was found between dental anxiety (MDAS) and gender (Chi-square test, *p* < 0.05). The median MDAS value in women was higher than that in men in the paired comparisons (Mann–Whitney U test, *p* < 0.05; Table 3).

**Table 3:** Test statistics and *p*-values for the comparison of the level of dental anxiety according to the examined age and gender variables in the study (*N* = 500)

**Variable**	**n (%)**	**Statistical Test**	*p*
**Age**		Kruskal–Wallis X^2^ = 14.197	0.007*
˂ 20^ab^	16 (3.2)		
20–34^b^	182 (36.4)		
35–49^ab^	169 (33.8)		
50–64^a^	107 (21.4)		
65–79^ab^	23 (4.6)		
> 79^ab^	3 (0.6)		
**Gender**		Mann–Whitney U U = 20164.500	≤ 0.001**
Female	276 (55.2)		
Male	224 (44.8)		

^*^Kruskal–Wallis test (X^2^)

^**^Mann–Whitney U test (U): test statistics. The bold number indicates statistical significance, *p* < 0.05. Different lowercase superscripts show a statistically significant difference between the columns and variable groups

On the one hand, patients with no regular income had higher MDAS median values than those with regular income. On the other hand, the median MDAS value of persons with health insurance was higher than that of those without health insurance. In addition, the median value of the dental anxiety score (MDAS) of individuals who visited the dentist with a companion was higher than that of those who visited the dentist without a companion. A statistical difference was found among dental anxiety levels according to the reason for dental visits (Mann–Whitney U, *p* < 0.05). The median MDAS values of individuals who did not remember the reason for the dental visit were higher than the median value of those who visited the dentist for routine control (Kruskal–Wallis test, *p* < 0.05; Table 4).

**Table 4:** Test statistics and *p*-values of the relationship between dental anxiety level and patient variables (*N* = 500)

**Variable**	***N* (%)**	**Statistical Test**	***p***
**Education level**		Chi-square x^2^ = 7.079	0.314***
Uneducated	8 (1.6)		
Primary school	151 (30.2)		
High school	146 (29.2)		
University	195 (39.0)		
**Marital status**		Chi-square x^2^ = 2.865	0.581***
Single	143 (28.6)		
Married	336 (67.2)		
Divorced	21 (4.2)		
**Having any children**		Chi-square x^2^ = 0.002	0.999***
No	168 (33.6)		
Yes	332 (66.4)		
**Smoking**		Chi-square x^2^ = 0.002	0.130***
No	294 (58.8)		
Yes	145 (29)		
Smoked in the past	61 (12.2)		
**Monthly regular income**		Mann–Whitney U U = 23,090.000	0.001**
No	193 (38.6)		
Yes	307 (61.4)		
**Health insurance**		Mann–Whitney U U = 23,090.00	≤0.001**
No	43 (8.6)		
Yes	457 (91.4)		
**Place of residence**		Chi-square x^2^ = 8.245	0.083***
City	335 (67,0)		
Township	114 (22.8)		
Village	51 (10.2)		
**Systemic disease**		Chi-square x^2^ = 8.245	0.142***
No	406 (81.2)		
Yes	94 (18.8)		
**Drug use**		Chi-square x^2^ = 4.047	0.132***
No	423 (84.6)		
Yes	77 (15.4)		
**Bruxism**		Chi-square x^2^ = 3.187	0.203***
No	312 (62.4)		
Yes	188 (37.6)		
**Nail biting habit**		Chi-square x^2^ = 4.068	0.131***
No	449 (89.8)		
Ye**s**	51 (10.2)		
**Foreign object biting habit**		Chi-square x^2^ = 0.262	0.877***
No	486 (97.2)		
Yes	14 (2.8)		
**Prosthetic use**		Chi-square x^2^ = 1.298	0.532***
No	344 (68.8)		
Yes	156 (31.2)		
**Ever visited a dentist before**		Chi-square x^2^ = 0.115	0.994***
No	12 (2.4)		
Yes	488 (97.6)		
**Visiting the dentist with a companion person**		Mann–Whitney U U = 23,881.000	≤0.001**
No	297 (59.4)		
Yes	203 (40.6)		
**Frequency of dental visit**		Kruskal–Wallis X^2^ = 0.355	0.837*
Rare	74 (14.8)		
When having a complaint	384 (76.8)		
Regularly	42 (8.4)		
**Reason for a dental visit**		Kruskal–Wallis X^2^ = 8.131	0.043*
Pain^ab^	135 (27)		
Treatment/appointment^ab^	314 (62.8)		
Routine check up^a^	33 (6.6)		
Do not remember^b^			

^*^Kruskal–Wallis test (X^2^)

^**^Mann–Whitney U test (U)

^***^Chi-square test (x^2^): test statistics. Bold numbers indicate statistical significance, *p* < 0.05. Different lowercase superscripts show a statistically significant difference between the columns and variable groups

No statistically significant relationship was found between the dental anxiety score (MDAS) of individuals and the educational status, marital status, smoking habit, and frequency of dental visits (Chi-square test, *p* > 0.05; Table 5).

**Table 5:** Test statistics and *p*-values of the relationship between dental anxiety levels and patient variables (*N* = 500)

**Variable**	***N* (%)**	**Statistical Test**	p***
**Tooth brushing habit**		Chi-square x^2^ = 4.806	0.09
No	23 (4.6)		
Yes	477 (95.4)		
**Brushing frequency**		Chi-square x^2^ = 8.384	0.591
None	23 (4.6)		
Once a day	274 (54.8)		
≥2 per day	172 (34.4)		
Once a week	22 (4.4)		
>1 per week (2–6 times)	5 (1)		
>1 per month (2–6 times)	4 (0.8)		
**Using toothpaste**		Chi-square x^2^ = 1.263	0.532
Not brushing teeth	23 (4.6)		
No	5 (1)		
Ye**s**	472 (94.4)		
**Using toothpaste with fluoride**		Chi-square x^2^ = 5.605	0.231
Not brushing teeth	23 (4.6)		
No	16 (3.2)		
Yes	197 (39.4)		
Does not know	264 (52.8)		
**Tooth brushing time**		Chi-square x^2^ = 8.733	0.189
1–2 minutes	240 (48)		
2–5 minutes	179 (35.8)		
>5 minutes	34 (6.8)		
Billions	47 (9.4)		
**Type of toothbrush**		Chi-square x^2^ = 9.905	0.129
Hard	47 (9.4)		
Moderate	204 (40.8)		
Soft	71 (14.2)		
Does not know	155 (31)		
**Products used in oral care**		Chi-square x^2^ = 0.807	0.668
Toothbrush	477 (95.4)		
Toothpick	56 (11.2)		
Dental floss	62 (12.4)		
Miswak	4 (0.8)		
Other	6 (1.2)		
**Eating meals regularly**		Chi-square x^2^ = 0.367	0.832
No	113 (22.6)		
Yes	387 (77.4)		
**Consuming food between meals**		Chi-square x^2^ = 3.848	0.146
No	89 (17.8)		
Yes	411 (82.2)		
**Frequency of sweet food/drink consumption**		Chi-square x^2^ = 6.616	0.761
Never	17 (3.4)		
Once a month	53 (10.6)		
Once a week	122 (24.4)		
>1 per week	144 (28.8)		
Once a day	125 (25)		
>1 per day	39 (7.8)		
**Eating before sleeping**		Chi-square x^2^ = 1.318	0.517
No	352 (70.4)		
Yes	148 (29.6)		

^***^Chi-square test (x^2^): test statistics, *p* > 0.05

A statistically significant relationship was found between dental anxiety (MDAS) and the total number of teeth in individuals. The dental anxiety rate was higher in participants with 1–9 teeth than in participants with more than 20 teeth (Chi-square test, *p* = 0.05). The MDAS values of individuals who experienced pain at the first and current filling treatments were higher than those who did not experience any pain (Mann–Whitney U test, *p* < 0.05). Individuals who postponed current appointments had higher MDAS values than those who did not. In addition, the median MDAS value of individuals who received local anaesthesia during treatment was higher than those who were not applied (Mann–Whitney U test, *p* < 0.05). Patients with self-reported bad sensation at the current filling treatment had the highest median MDAS value in the present study compared with the other self-reported sensation groups (Kruskal–Wallis test, *p* < 0.05; Table 6).

**Table 6:** Test statistics and *p*-values of the relationship between dental anxiety level and variables of the participants (*N* = 500)

**Variable**	***N* (%)**	**Statistical Test**	***p***
**Total number of teeth**		Chi-square x^2^ = 9.510	0.050***
1–9	7 (1.4)		
10–19	44 (8.8)		
≥20	449 (89.8)		
**Had any dental filling experience**		Chi-square x^2^ = 0.809	0.667***
No	11 (2.2)		
Yes	489 (97.8)		
**First dental filling**		Chi-square x^2^ = 9.558	0.145***
<12	141 (28.2)		
12–18 years old	134 (26.8)		
>18 years old	111 (22.2)		
Unclear	114 (22.8)		
**Pain during first dental filling**		Mann–Whitney U U = 12571.000	≤0.001**
No	334 (66.8)		
Yes	166 (33.2)		
**Pain during current filling**		Mann–Whitney U U = 4929.500	≤0.001**
No	462 (92.4)		
Yes	38 (7.6)		
**Sensation of the current filling experience**		Kruskal–Wallis X^2^ = 177.224	≤0.001*
Bad^a^	76 (15.2)		
Not bad^b^	224 (44.8)		
Good^c^	200 (40)		
**Postponed current appointment**		Mann–Whitney U U = 5250.500	≤0.001**
No	457 (91.4)		
Yes	43 (8.6)		
**Had any restoration on tooth surfaces**		Mann–Whitney U U = 25486.500	0.861**
No	335 (71)		
Yes	145 (29)		
**Application of local anesthesia**		Mann–Whitney U U = 12847.000	0.014**
No	427 (85.4)		
Yes	73 (14.6)		
**Who performed the treatment?**		Mann–Whitney U U = 18472.500	0.151**
Intern	398 (79.6)		
Lecturer	102 (20.4)		
**Restorative material used**		Chi-square x^2^ = 2.742	0.602***
Amalgam	1 (0.2)		
Composite	497 (99.4)		
Glass ionomer	2 (0.4)		

^*^Kruskal–Wallis test (X^2^),

^**^Mann–Whitney U test (U)

^***^Chi-square test (x^2^): test statistics. Bold numbers indicate statistical significance, *p* < 0.05. Different lowercase superscripts show a statistically significant difference between the columns and variable groups

## Relationship between Dental Anxiety (MDAS) and DMFT, DMFS, and GIs

No statistically significant relationship was found between the MDAS and DMFT and DMFS indexes in the individuals (Spearman correlation analysis, *p* > 0.05; Table 7). Furthermore, no statistically significant relationship was observed between the dental anxiety levels (MDAS) of patients and GI scores (Chi-square test, *p* > 0.05; Table 8).

**Table 7:** Test statistics on the relationship between dental anxiety level and decayed, missing, or filled teeth (DMFT) and decayed, missing or filled surfaces (DMFS) indices of the study participants (*N* = 500)

	**Spearman Correlation**	***p***
	**Coefficient**	
Decayed teeth	0.027	0.546
Missing teeth	−0.082	0.067
Filled teeth	0.033	0.463
**DMFT Value**	−0.055	0.219
Decayed tooth surface	0.035	0.431
Missing tooth surface	−0.074	0.097
Filled teeth surface	0.049	0.277
**DMFS Value**	−0.061	0.174

Spearman correlation test, *p* > 0.05

**Table 8:** Test statistics (*N* = 500) of the relationship between dental anxiety level and periodontal status according to gingival index (Löe, 1967) (*N* = 500)

**Score**	***N* (%)**	**Statistical Test**	p***
0 (*no inflammation*)	8 (1.6)	Chi-square x^2^ = 3.516	0.742
1 (*mild inflammation*)	274 (54.8)		
2 (*moderate inflammation*)	209 (41.8)		
3 (*severe inflammation***)**	9 (1.8)		

^***^Chi-square test (x^2^): test statistics, *p* > 0.05

## Discussion

In the present study, MDAS was used to assess anxiety levels because it is a concise, easy-to-use, and accepted scale [18]. Dou et al. found that the mean for the MDAS score is 14.17 higher than our scores [25]. A study in South India that included 1,148 adults has reported that the mean dental anxiety level is 10.4, which was consistent with our findings [26]. The median value of MDAS is 9.00, indicating that most participants in the current study exhibited less anxiety [25].

In the literature, traumatic dental experiences, personal characteristics, gender, age and education levels may affect the dental anxiety level of patients [26–30]. Compared with the current study, a few studies in the literature have shown an opposite association between age and dental anxiety levels [27, 31, 32]. A study has shown that the anxiety level of patients in the age group of 20–39 years was higher than in younger and older groups, which was in accordance with our findings [33]. In the current study, although no statistical difference was found between all age groups in terms of dental anxiety level, the median value of MDAS of patients in the age group of 20–34 years was higher than the median value of MDAS in the age group of 50–64 years. This result may be because over time and with age, enhanced exposures have allowed the patients to indulge in the treatment and have less anxiety.

Gender differences can be a determining factor in dental anxiety levels [34–36]. Previous studies have reported that during dental treatment, women experience more anxiety than men, which was in accordance with the current study [3, 34–38]. Although no statistical difference according to gender was found, the level of anxiety in women is higher than in men in one study [39]. Notably, the difference in dental anxiety between the sexes may be due to structural and functional alterations in the brain and hormonal changes. Furthermore, this difference may depend on the higher stress levels and phobias of women compared with men in the present study [37].

A few studies have examined the relationship between income levels and anxiety levels [14, 38]. Tunç et al. found no significant relationship between MDAS scores and income levels, which was in contrast to our findings [14]. Melchior et al. found that individuals in low-income families are highly prone to anxiety and depression, which was in accordance with our findings [40]. This study found a statistically significant relationship between anxiety levels and monthly income. Furthermore, in this study, those with a regular monthly income had lower anxiety scores than those without a regular monthly income.

High levels of dental anxiety can be considered the primary reason for tooth loss, treatment cancellation or missed dental appointments [41]. In support of our findings, previous studies have reported that patients with anxiety about visiting the dentist tend to postpone their treatment unless during an emergency [41–44]. Furthermore, people with dental anxiety have irregular visits to dentists [3, 25, 45]. Restorative treatments or tooth loss management may be considered with regular dental attendees; however, patients with dental anxiety may perceive that dentists only merely offer symptomatic care for their declining oral health rather than a comprehensive treatment [3]. Haggling et al. reported that individuals with high anxiety have fewer teeth than those with lower anxiety, which supported our results [46]. The anxiety rate of patients with 1–9 teeth was higher than that of the patients with more than 20 teeth in the current study. Unfortunately, this may be due to delayed dental visits, and the existing teeth often become incurable or untreatable anymore [47].

Previous studies, as well as the current study, have reported a significant relationship between dental anxiety and pain [25, 41]. Dou et al. reported that during the most recent dental examination, patients who felt a high rate of pain tended to have high anxiety [25]. Patients who desire the administration of anaesthesia during treatment have signs of dental fear or anxiety [41]. In our study, similar to the findings of the previous study, patients who experienced pain during filling had higher anxiety rates than patients without pain. Furthermore, the dental anxiety rate of individuals who received local anaesthesia during treatment was higher than that of those without any anaesthesia. This was considered to provide effective treatment in dentally anxious patients; particularly in dentally phobic individuals, more effective local anaesthesia and anti-anxiety strategies should be developed in the future for dental treatment.

In the literature, several studies have compared dental anxiety levels with the DMFT index [48, 49]. A study has found a significant and inversely proportional relationship between the number of filled teeth and anxiety, which was in contrast to our findings. Zinke et al. found a significant relationship between dental anxiety levels and the DMFS index. In addition, the rate of hard tissue destruction due to caries is higher in individuals with high dental anxiety than in individuals with low dental anxiety levels [50]. Folayan et al. also showed that the number of decayed teeth in patients with high anxiety levels is higher than in those with low anxiety levels [51]. Some studies have reported no significant relationship between dental anxiety and the DMFT index, which was consistent with our results [36, 48, 49, 52]. A previous study has reported no statistically significant relationship between the number of teeth extracted (MT) and the number of filled teeth (FT) and dental anxiety, which was in accordance with our results [36]. The present study found no significant relationship between MDAS and DMFT and DMFS scores, which are the indices associated with dental caries prevalence employed in previous studies.

No correlation exists between the anxiety level of patients and periodontal parameters in several studies [36, 53, 54]. Similarly, no relationship was found between MDAS and GI, which is a periodontal parameter in the present study. The fact that the research group consisted of individuals who applied to the Faculty of Dentistry for treatment can be considered a limitation of the present study. A small sample group in the study cannot be entirely representative of the community regarding dental anxiety levels. In addition, periodontal evaluation performed with only the gingival index and not using the plaque index can be considered another limitation of the study. The gingival health level of individuals could not be completely revealed with a single periodontal parameter in the study.

## Conclusion

The findings of the study indicated that dental anxiety negatively affected the dental visiting habits of patients. In this cross-sectional study, patients with dental anxiety had more missing teeth and more frequently postponed their dental appointments. Further studies on the relationship between dental anxiety and oral health are necessary to ensure the benefit of dental services and to create an effective treatment for persons with dental anxiety.

## Data Availability

The datasets used for the current study can be made available from the corresponding author on reasonable request.

## Abbreviations

MDAS: Modified dental anxiety scale
DMFT: Decayed, missed, filed teeth
DT: Decayed Teeth
MT: Missed teeth
FT: Filled teeth
DMFS: Decayed, missed, filled teeth surface
DS: Decayed teeth surface
MS: Missed filled surface
FS: Filled teeth surface
GI: Gingival index
WHO: World Health Organization
